# Low-energy tetrahedral networks for carbon and silicon from (2+1)-regular bipartite-like graphs

**DOI:** 10.1107/S2052252525005810

**Published:** 2025-07-29

**Authors:** Yalan Wei, Shifang Li, Xizhi Shi, Chaoyu He

**Affiliations:** a School of Physics and Optoelectronics Xiangtan University Xiangtan411105 People’s Republic of China; b Center for Quantum Science and Technology Shanghai University Shanghai200444 People’s Republic of China; Tsinghua University, China

**Keywords:** 3D 4-coordinate networks, 2D (2+1)-regular bipartite-like graphs, random group and graph theory, superhard carbon, quasi-direct band gap silicon

## Abstract

Post-graphite in cold-compression [Mao *et al.* (2003). *Science***302**, 425–427] has attracted widespread research interest in condensed matter physics. Subsequently, many low-energy carbon phases, such as M-carbon, W-carbon and Z-carbon, have been proposed as structural candidates. Based on graph theory, we found that these 4-coordinated post-graphite candidates can not only be decomposed into 3-regular graphitic graphs but also yield (2+1)-regular graphs in a non-graphitic manner from different decomposition directions. This inspires a general idea of generating 3D 4-coordinate networks based on 2D (2+1)-regular bipartite-like graphs, which can be efficiently generated by a random method combined with group and graph theory (RG2). Associated with such a general graph-based method, a large number of 4-coordinate networks have been discovered and investigated by first-principles calculations as potential carbon/silicon phases. Most are confirmed as low-energy carbon/silicon phases and identified as direct or quasi-direct band gap semiconductors. Two complex configurations, *Pbam*48 and *Pbam*40, show energetic stabilities exceeding or comparable to the previously proposed *Pbam*24. They are further confirmed to be dynamically and mechanically stable phases as carbon/silicon. As carbon phases, *Pbam*48 and *Pbam*40 are superhard insulators with quasi-direct band gaps of 5.622 and 5.890 eV, and hardness values of 85.352 and 85.558 GPa, respectively. Their X-ray diffraction (XRD) results can largely explain the experimental XRD patterns of cold-compressed graphite. As silicon allotropes, *Pbam*48 and *Pbam*40 have quasi-direct band gaps of 1.386 and 1.451 eV, respectively, making them potential solar cell absorber materials.

## Abstract 2

This study identifies carbon crystal structures as 4-degree quotient graphs and proposes a novel approach for generating 3D 4-coordinate networks using 2D (2+1)-regular bipartite-like graphs. Through a random group and graph theory (RG2) method, we discover 509 new structures, including two low-energy phases (*Pbam*48 and *Pbam*40) that exhibit exceptional stability and potential applications as superhard carbon and quasi-direct band gap silicon materials in mechanical processing and solar photovoltaic technologies.

## 1. Introduction

Crystal structure prediction is one of the most important tasks in computational condensed matter physics and materials sciences (Woodley & Catlow, 2008 ▸; Oganov *et al.*, 2019 ▸). The theoretical understanding of the fundamental properties of any crystalline material is inseparable from its crystal structure (Hawfhorne, 1990 ▸), which determines almost all of the physical and chemical properties of a crystalline material. For example, the carbon atoms arranged in different types of periodic patterns in 3D space give rise to two entirely different forms: the superhard insulating transparent diamond and the soft conductive black graphite. Carbon-based materials are indeed a testing ground for various new crystal structure prediction methods (Glass *et al.*, 2006 ▸; Lonie & Zurek, 2011 ▸; Pickard & Needs, 2011 ▸; Wang *et al.*, 2012 ▸; Takagi *et al.*, 2017 ▸; Shi *et al.*, 2018 ▸; Wang *et al.*, 2010 ▸; Strong *et al.*, 2004 ▸; Zhou & Zeng, 2012 ▸), as carbon has a rich variety of bonding manners (*sp*, *sp*^2^ and *sp*^3^) to form a wide range of isomers. In addition to graphite and diamond, it has been confirmed that elemental carbon can form many other experimentally realized isomers, such as graphite (Niu *et al.*, 2012 ▸; Novoselov *et al.*, 2004 ▸), carbon nanotubes (Iijima, 1991 ▸), fullerenes (Kroto *et al.*, 1985 ▸), V-carbon (Yang *et al.*, 2017 ▸), T-carbon (Sheng *et al.*, 2011 ▸; Zhang *et al.*, 2017 ▸), simple cubic carbon (Yamada, 2003 ▸; He *et al.*, 2017 ▸), super-size cubic and hexagonal carbons (Whittaker & Wolten, 1972 ▸; El Goresy & Donnay, 1968 ▸; Whittaker & Kintner, 1969 ▸; El Goresy *et al.*, 2003 ▸; Liao *et al.*, 2023 ▸), and some post-graphite phases discovered in cold-compression processes (Mao *et al.*, 2003 ▸). Many carbon isomers have been theoretically predicted (*SACADA*; https://sacada.sacada.sctms.ru/) either to analyse experimentally synthesized carbon crystals or to design new carbon materials with exceptional properties. For example, the superhard bct-C4 (Umemoto *et al.*, 2010 ▸), M-carbon (Li *et al.*, 2009 ▸), Z-carbon (Amsler *et al.*, 2012 ▸; Zhao *et al.*, 2011 ▸), W-carbon (Wang *et al.*, 2011 ▸) and S-carbon (He *et al.*, 2012 ▸) proposed for explaining the cold-compressed graphite (Mao *et al.*, 2003 ▸). The XRD, hardness and electronic structures of these candidate structures can all, to some extent, help us to understand the experimental results (Mao *et al.*, 2003 ▸), and all their structural geometries can be derived from *sp*^2^ to *sp*^3^ phase transitions of graphite through intralayer fluctuations and interlayer hybridizations.

Different perspectives often help us gain a more comprehensive understanding of things and open up new ideas and approaches to explore and solve problems. For example, crystal structures can be considered as quotient graphs from a mathematical view and this provides great convenience for crystal structure prediction (Strong *et al.*, 2004 ▸; Zahariev *et al.*, 2006 ▸; Winkler *et al.*, 2001 ▸) and the development of machine learning potentials (Wang *et al.*, 2023 ▸; Li *et al.*, 2023 ▸; Zhong *et al.*, 2023 ▸; Xie & Grossman, 2018 ▸). This work extends beyond conventional methodologies such as direct structural search or simulating graphite compression pathways for synthesizing superhard post-graphite phases (Yang *et al.*, 2017 ▸; Sheng *et al.*, 2011 ▸; Umemoto *et al.*, 2010 ▸; Li *et al.*, 2009 ▸; Amsler *et al.*, 2012 ▸; Zhao *et al.*, 2011 ▸; He *et al.*, 2012 ▸). We examine the previously proposed carbon crystal structures as isomorphic to 4-degree quotient graphs from a graph-theoretical perspective. We find that, in addition to being decomposed into graphite phases isomorphic to 3-degree quotient graphs (Niu *et al.*, 2012 ▸; Zhou & Zeng, 2012 ▸; He *et al.*, 2012 ▸), most of them can also be decomposed from another perspective into non-graphite phases corresponding to (2+1)-regular bipartite-like graphs. This inspires a general idea of generating 3D 4-coordinate networks by searching the 2D (2+1)-regular bipartite-like graph with up and down sequence of D:DDU and U:DUU. Associated with the random method combining group and graph theory (RG2) (Shi *et al.*, 2018 ▸) and first-principles calculations, a scheme and algorithm are designed to implement this idea. In addition to identifying previously proposed configurations, we discovered numerous new low-energy structures as candidates for carbon and silicon. The two lowest energy ones are further confirmed as dynamically and mechanically stable superhard carbon and quasi-direct band gap silicon, which are potential materials for applications in the mechanical processing industry and the solar photovoltaic industry, respectively. These results effectively demonstrate the reliability and feasibility of this graph-based methodology proposed for crystal structure prediction.

## 2. Graph theory definitions

**Figure 1:**
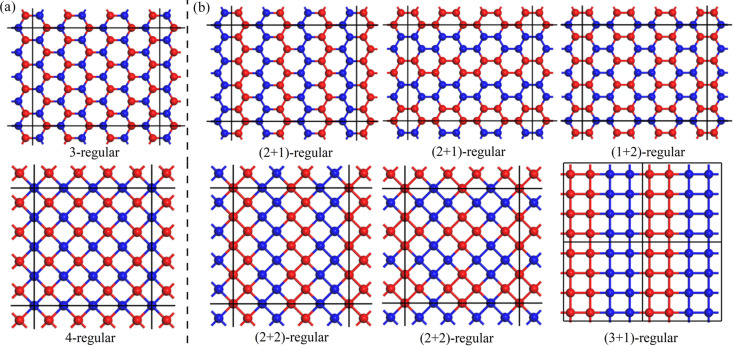
(*a*), (*b*) Selected examples for 3-regular and 4-regular bipartite graphs, and their corresponding (*n*+*m*)-regular bipartite-like graphs, respectively.

Before presenting our results, we need to provide some basic definitions in graph theory. As shown in Fig. 1 ▸(*a*), two periodic bipartite lattices are selected as the three- and four-coordinated hexagonal and tetragonal lattices, respectively. As graphs *G* = (*V*, *E*), their vertex sets *V* can be divided into two subgroups of *V*_1_ (blue atom) and *V*_2_ (red atom), with *V*_1_ ∩ *V*_2_ = **∅** and *V*_1_ ∪ *V*_2_ = *V*. It is clear that each edge *e_ij_* ∈ *E* connects two vertices belonging to different subsets and each vertex *v_i_* has the same number of edges (*n*) connected to it. In graph theory, they are referred to as *n*-regular bipartite graphs, in which *n*-regular and bipartite are two independent features of the graph. In our present work, an (*n*+*m*)-regular bipartite-like graph is defined as a *k*-regular graph (*k* = *m*+*n*) with two groups of vertexes *V*_1_ and *V*_2_, in which each vertex has *n* edges linking to neighbors in the same sets and *m* edges linking to neighbors in opposite sets. Fig. 1 ▸(*b*) shows some (*n*+*m*)-regular graphs obtained by reclassifying the vertexes in the given *k*-regular graphs in Fig. 1 ▸(*a*). It is evident that the (*n*+*m*)-regular bipartite-like characteristics will not change due to swapping the labels of the two parts of *V*_1_ and *V*_2_.

**Figure 2:**
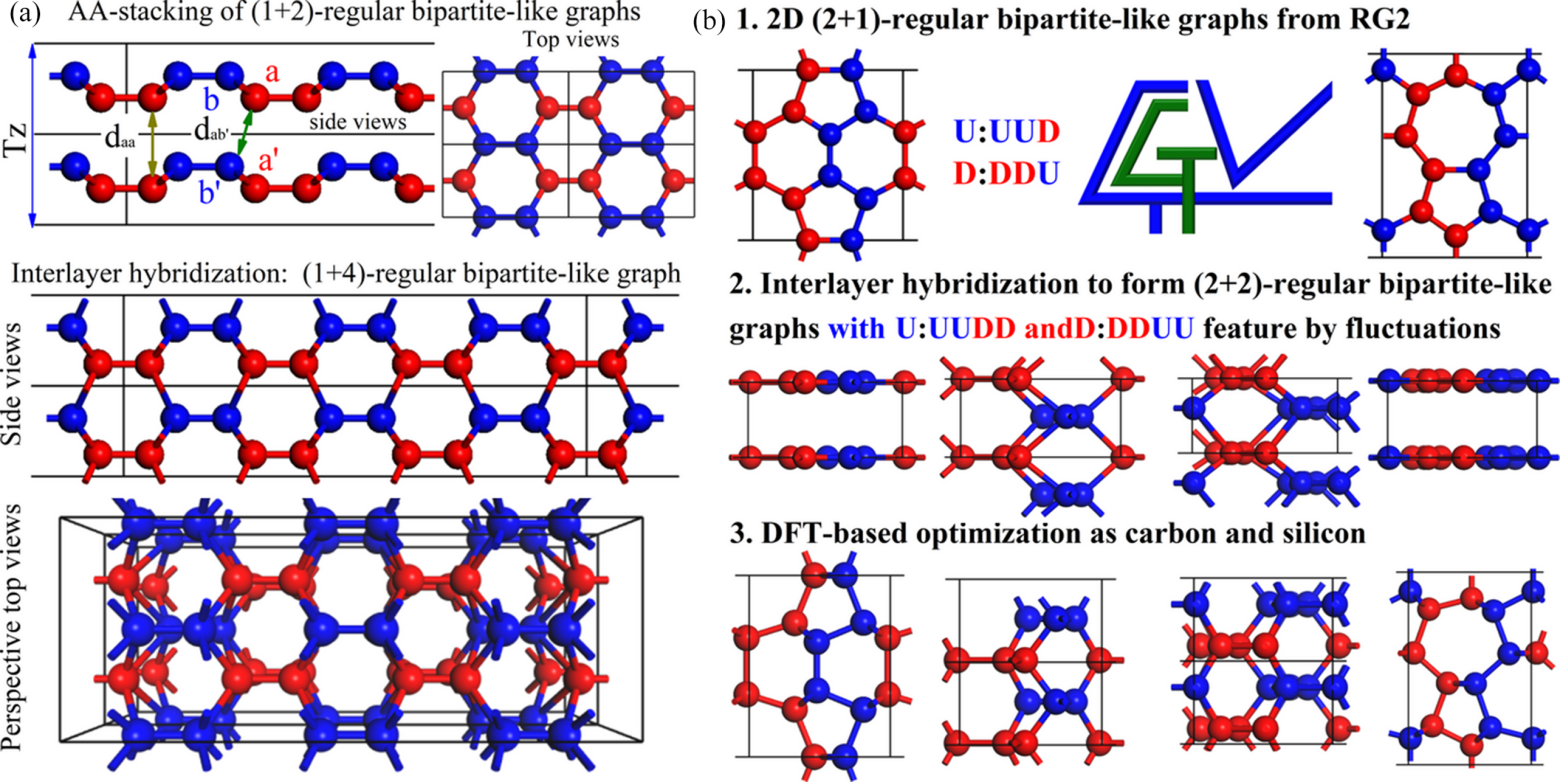
(*a*) Illustrative example of converting a 2D (*n*+*m*)-regular bipartite-like graph in AA stacking into a 3D (*n*+2*m*)-regular bipartite-like graph. (*b*) Schematic workflow leveraging RG2 to explore 4-regular carbon and silicon configurations.

In practical applications of condensed matter physics, the classification of these graph vertices can be visualized as the directions of magnetic moments, polarization or atomic fluctuation. As shown in Fig. 2 ▸(*a*), if we visualize the vertex classification of bipartite-like graphs (lattice) as the up (blue balls) and down (red balls) fluctuations in the 2D graphs (lattice), new 3D graphs can be constructed by a *z*-direction vertical stacking. The *z*-direction fluctuations of (vertexes) atoms are introduced to reduce the interlayer (inter-graph) distance for forming new connections. In the AA-stacked bilayers, atoms aligned directly along the *z* direction in the two layers exhibit the same fluctuation pattern. Their distance in the *z* direction is exactly half of the *z*-direction periodicity (*Tz*), which also corresponds to the minimum interlayer distance *d*_*aa*_ (*d*_*bb*_) between atoms of the same type. The nearest interlayer neighbors of a certain type of atom (*a*) from the opposite set are the projections (*b*′) of its intralayer neighbors (*b*) onto the next layer (in distance of *d*_*ab*′_). Under a certain *z*-direction periodicity and fluctuation amplitude, distance *d*_*ab*′_ can be always smaller than *d*_*aa*_. Thus, the newly added neighbors for any type of atoms (*a*) are always of the opposite type (*b*). That is to say, the combination of AA-stacked (*n*+*m*)-regular bipartite-like graphs will result in 3D (*n*+2*m*)-regular bipartite-like ones, given as a example in Fig. 2 ▸(*a*) for stacking a (1+2)-regular bipartite-like graph to a 3D (1+4)-regular one.

If we consider another stacking manner of AB, where B is defined as an adjacent A to A with only an exchanged *z*-direction up and down sequence, shown in Fig. S1 of the supporting information, what will happen? In this situation, the adjacent atoms (*a* → *b*′; *b* → *a*′) in A and B always belong to different types of up and down. Under a certain *z*-direction periodicity and fluctuation amplitude, the adjacent atoms experience either converging or diverging fluctuations. The adjacent atoms with converging fluctuations (*a* → *b*′) become closer within the same period and form new nearest neighbors, while adjacent atoms with diverging fluctuations (*b* → *a*′) will form nearest-neighbor relationships with counterparts in adjacent periods. That is to say, in the combination of AB-stacked (*n*+*m*)-regular bipartite-like graphs, each type of atom will gain an additional neighboring atom of the opposite type, thereby forming an (*n*+*m*′)-regular graph with *m*′ = *m* + 1. The example provided in Fig. S1 shows that stacking 2D (1+2)-regular bipartite-like graphs in an AB manner will form a new 3D (1+3)-regular one. Another point that needs to be discussed is the general knowledge regarding the combination and decomposition of graphs. As we know, the sum of two numbers is unique, but there are infinitely many ways to split a number into the sum of two numbers. Similarly, when we decompose these high-degree 3D graphs into lower-degree 2D graphs, the decomposition is not unique. Those ruled results regarding graph combination and decomposition are useful for guiding us in solving a number of specific problems.

## 3. Results

To be specific, let us examine the crystal structure of carbon with 4-coordinate *sp*^3^ hybridization from the perspective of graph theory. Previous studies have noted that a class of these carbon crystals can be generated by introducing various in-plane fluctuations and interlayer hybridizations into graphite. In other words, they can be decomposed into multiple graphite layers with distinct fluctuation patterns, effectively transforming a 3D 4-regular graph into several 2D 3-regular graphs. As illustrated in Fig. S2, the resulting 2D 3-regular graphs for a given 3D 4-regular graph are not always bipartite or bipartite-like. For example, the 3D S-carbon can be decomposed into equal graphite layers with hybridized (0+3) and (2+1) features. And the results show that M-carbon (W-carbon) can be decomposed into two different graphite layers with pure (0+3) and (2+1) bipartite-like features, respectively. We found that under this decomposition scheme into graphite, it is challenging to identify a unified pattern for interlayer stacking and interlayer hybridization. However, if these 3D structures are decomposed into non-graphite layers along a different crystal direction, they (M-carbon, W-carbon and S-carbon) all exhibit AA stacking, and the fluctuation pattern of each layer can be uniformly described as (2+1)-regular bipartite-like graphs. This is consistent with the previously discussed stacking patterns that AA-stacking of (2+1)-regular bipartite-like graphs will result in 3D (2+2)-regular bipartite-like ones. These findings imply a potential method for generating 3D 4-coordinate networks by stacking 2D (2+1)-regular bipartite-like graphs. And the potential 2D (2+1)-regular bipartite-like graphs with up and down sequence of D:DDU and U:DUU can be easily generated by the random method combining with group and graph theory as implemented in our previously developed RG2 code (He *et al.*, 2018 ▸; Yin *et al.*, 2019 ▸; Liao *et al.*, 2021 ▸; Zhang *et al.*, 2024 ▸).

As shown in Fig. 2 ▸(*b*), the rules discussed above can help us systematically search for potential 3D carbon or silicon structures in three steps. In the first step, 2D bipartite-like graphs that satisfy the (2+1)-regularity condition are systematically customized by RG2 code. In the second step, compress the periodicity *Tz* to approximately twice the C—C bond length and unify the fractional *z* coordinates of the two types of atoms to 0 and 1/2, respectively. Then introduce interlayer hybridization based on the nearest-neighbor association principle, resulting in a translation from AA-stacked 2D (2+1)-regular bipartite-like graphs to corresponding 3D (2+2)-regular bipartite-like ones. Finally, these 3D (2+2)-regular bipartite-like graphs are treated as 4-regular graphs as potential carbon and silicon crystals by DFT-based first-principles calculations on the structures, stabilities and fundamental properties. Please note that, as discussed before, AB-stacking of 2D (1+2)-regular bipartite-like graphs will result in 3D (1+3)-regular bipartite-like ones, which are also potential 4-regular graphs for carbon and silicon. However, this approach will inevitably result in high-energy four-membered ring structures, which are therefore not considered in our current work. As shown in Fig. S3, some 3-regular graphs are classified as (0+3)-regular (*a*), (1+2)-regular (*b*) and (2+1)-regular (*c*) bipartite-like graphs discovered by RG2.

In the RG2 search process, we considered random cells with lattice constants of 2 Å < *a*, *b* < 30 Å, *c* = 20 Å, 30° < α, β < 150° and γ = 90°. Two types of nodes labeled as nitrogen (N → 7) and oxygen (O → 8) are randomly projected in the cells with application of random symmetry operations of the layer groups (Nos. 1–80). For (2+1)-regular bipartite-like graphs, the neighboring relationships are described as ‘addbondlist 7 7 7 8’ and ‘addbondlist 8 8 8 7’ in RG2 for N (N:NNO) and O (O:OON) atoms, respectively. The structures meeting the conditions of N:NNO and O:OON will be further optimized by RG2 to make the bond lengths (angles) as close as possible to 1.426 Å (120°). All the surviving structures will be converted into 4-coordinate carbon and silicon structures for further optimization through the DFT-based first-principles calculations using the widely used *VASP* code (Kresse & Furthmüller, 1966 ▸). The PBE version generalized gradient approximation was applied in combination with projector augmented wave (PAW) (Blöchl, 1994 ▸; Kresse & Joubert, 1999 ▸) potentials to provide accurate descriptions of electron–ion interactions. The plane-wave cutoff energy was established at 500 eV, with the electronic self-consistency convergence set to 10^−5^ eV and the force convergence threshold fixed at 0.01 eV Å^−1^. The Brillouin zone was sampled with a sufficiently dense grid (spacing less than 0.26 × 2π Å^−1^) to ensure accuracy. To evaluate the dynamical and mechanical stabilities of the two most stable structures, *Pbam*48 and *Pbam*40, density functional perturbation theory (DFPT) (Wu *et al.*, 2005 ▸) was employed to calculate their vibrational spectra and elastic constants. The widely used *PHONOPY* (Togo & Tanaka, 2015 ▸) package was used to compute the phonon dispersion relations. Table S6 of the supporting information includes detailed calculation parameters.

**Figure 3:**
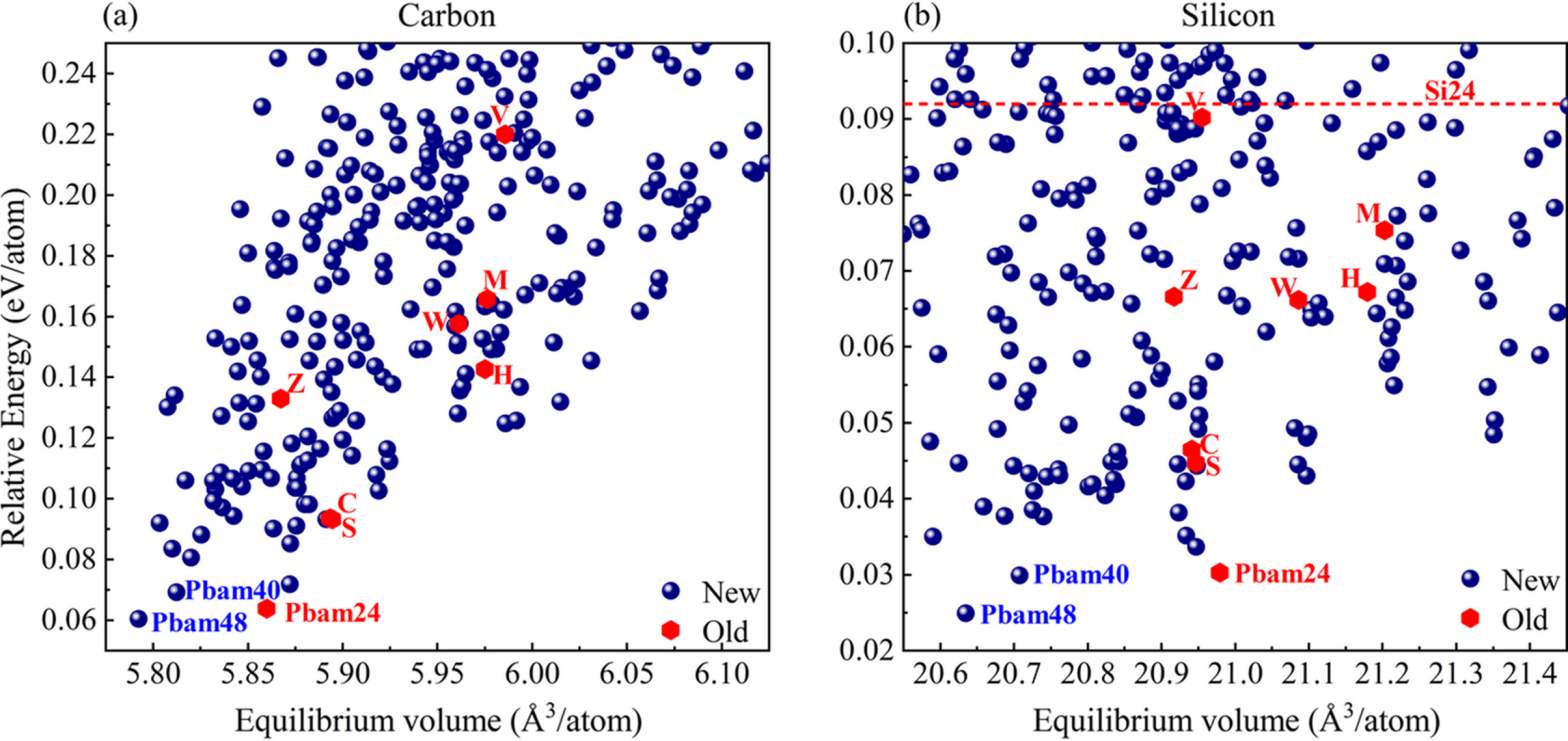
Scatter plots of the relative average energies (eV per atom) versus equilibrium volumes (Å^3^ per atom) of the potential (*a*) carbon and (*b*) silicon phases calculated from PBE functionals. Some well known old phases marked as red hexagons are selected for comparison.

From the results shown in Fig. 3 ▸, we identified a total of 509 non-equivalent 4-regular graphs as potential candidates for *sp*^3^-hybridized carbon and silicon structures. For carbon (silicon), those structures with energies approximately 0.30 eV per atom (and 0.15 eV per atom) higher than cubic diamond (cubic silicon) were selected and displayed as scatter plots of relative total energy (eV per atom) versus average volume (Å^3^ per atom). Notably, many previously proposed low-energy allotropes, such as M-carbon (Li *et al.*, 2009 ▸), Z-carbon (Amsler *et al.*, 2012 ▸), W-carbon (Wang *et al.*, 2011 ▸), H-carbon (He *et al.*, 2012 ▸), S-carbon (He *et al.*, 2012 ▸), C-carbon (Li *et al.*, 2012 ▸), V-carbon (Yang *et al.*, 2017 ▸) and *Pbam*24 (Mujica *et al.*, 2015 ▸) were rediscovered in our search and they are highlighted as red hexagons for comparison. From a thermodynamic perspective, using energy as a criterion, our search reveals numerous novel carbon structures potentially synthesizable relative to experimentally realized V-carbon (Yang *et al.*, 2017 ▸). Similarly, for silicon, many new structures show potential for synthesis when compared with the experimentally synthesized Si-24 (Kim *et al.*, 2015 ▸). To balance reliability and computational efficiency, we adopted tight-binding parameter fitting under HSE06-based (Heyd *et al.*, 2003 ▸) band structures to enable high-throughput calculations and classification of the band structures for these potential carbon and silicon crystals. For silicon crystals, we directly utilized previously fitted tight-binding parameters (Su *et al.*, 2022 ▸), which have been demonstrated to possess good structural transferability. For carbon structures, we selected four fitting structures [CFS (Pickard & Needs, 2010 ▸), c-diamond, BC8 (Biswas *et al.*, 1987 ▸) and T12 (Zhao *et al.*, 2012 ▸)] and four validation structures [h-diamond, R8 (Piltz *et al.*, 1995 ▸), *Pbam*24 (Mujica *et al.*, 2015 ▸) and St12 (Rapp *et al.*, 2015 ▸)] to ensure the reliability of the parameters. The results are presented in Fig. S4 and the corresponding parameters are listed in Table S1. As summarized in Fig. S5, the distribution of carbon allotropes among the three types of band gap semiconductors – direct (D), quasi-direct (Q) and indirect – are 39, 182 and 288, respectively. In contrast, silicon, which has larger overlap radii and relatively weaker interactions, exhibits smaller band gaps compared with its carbon counterparts. Consequently, the number of direct and quasi-direct band gap phases increases to 57 and 298, respectively, while the number of indirect band gap phases decreases to 154. Note that, a quasi-direct band gap means that the smallest indirect band gap is less than the smallest direct band gap, with the difference being within 0.15 eV according to previous literature (Lee *et al.*, 2014 ▸).

**Figure 4:**
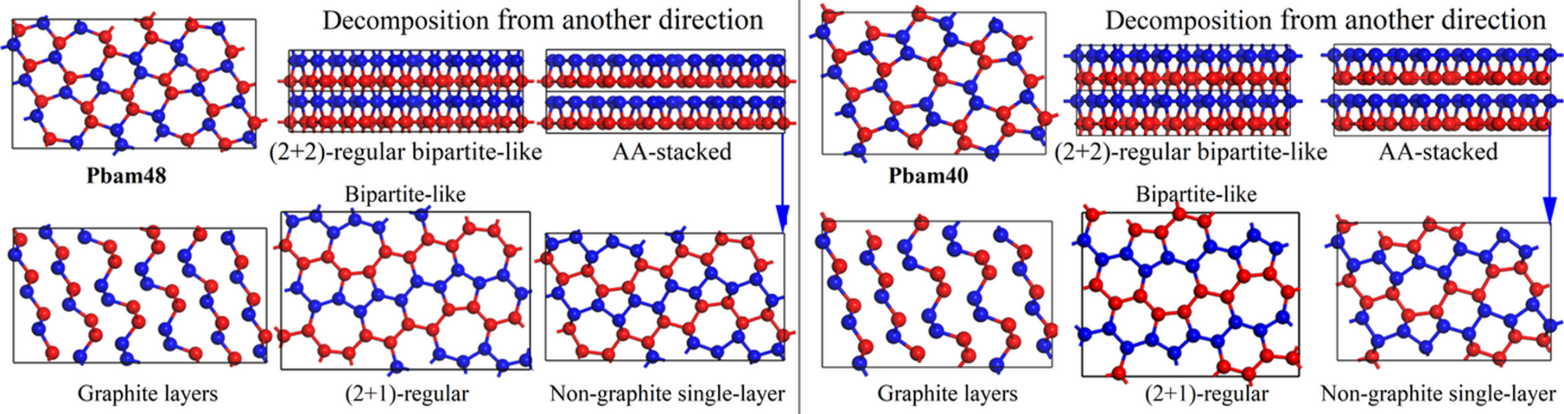
Optimized crystal structures of *Pbam*48 and *Pbam*40, as well as their direction-dependent decompositions to graphite or non-graphite layers.

Among these potential structural candidates, two exhibit exceptionally low energies, making them more stable than the previously proposed hypothetical allotrope *Pbam*24 (He *et al.*, 2018 ▸, Mujica *et al.*, 2015 ▸), though still less stable than the fully hexagonal configurations (Wei *et al.*, 2022 ▸) similar to c-diamond and h-diamond. These two structures captured our interest, prompting us to further investigate their structural characteristics, stability, mechanical properties and electronic properties. Their crystalline information, after optimization as carbon and silicon, are summarized in Tables S2 and S3 for interested researchers. As shown in Fig. 4 ▸, they are named *Pbam*48 and *Pbam*40 for their *Pbam* orthorhombic cells with 48 and 40 atoms per cell, respectively. The 4-regular quotient graphs of *Pbam*48 and *Pbam*40 can be decomposed into two different (2+1)-regular bipartite-like quotient graphs in an AA-stacked manner. Both these 2D (2+1)-regular bipartite-like graphs are obviously non-graphite ones with 5–6–7 member rings. It is interesting that *Pbam*48 can also be decomposed into 6 graphite layers, while *Pbam*40 contains 5 graphite layers with different intralayer up and down sequences. This indicates that both are potential carbon structures can be used for explaining the post-graphite phase synthesized in cold-compression processes. As shown in Fig. S6, they match the experimental XRD patterns (Mao *et al.*, 2003 ▸) more closely than the previously proposed M-carbon (Li *et al.*, 2009 ▸), Z-carbon (Amsler *et al.*, 2012 ▸), W-carbon (Wang *et al.*, 2011 ▸), H-carbon (He *et al.*, 2012 ▸) and S-carbon (He *et al.*, 2012 ▸), with many more accurate peak fittings. In addition to matching the main peaks, *Pbam*40 and *Pbam*48 within the 8.5–10.5° range also provide additional secondary peaks that help to explain the broadening observed in the experimental data. They also provide more secondary peaks in the range 15–16° for explaining the experimental XRD results in comparison with previously proposed ones (Li *et al.*, 2009 ▸; Amsler *et al.*, 2012 ▸; Wang *et al.*, 2011 ▸; He *et al.*, 2012 ▸; Mujica *et al.*, 2015 ▸). Moreover, they are both superhard, transparent insulators (as discussed below), which is also consistent with the experimental conclusions (Mao *et al.*, 2003 ▸).

The calculated energies of *Pbam*48 and *Pbam*40 are 60 and 69 meV per atom higher than that of c-diamond carbon, while they are 24 and 29 meV per atom higher than that of diamond silicon. They are nearly the most stable isomers in the carbon and silicon families (He *et al.*, 2018 ▸; Lee *et al.*, 2014 ▸), second only to the full six-ring configurations of hexagonal diamond (Wei *et al.*, 2022 ▸). To further confirm if they are dynamically and mechanically stable, we calculated their vibrational spectra and elastic constants, as the results shown in Fig. S7 and listed in Table S4, respectively. There are no imaginary frequencies appearing in the phonon bands. That is to say, as carbon or silicon, both *Pbam*48 and *Pbam*40 are dynamically stable phases under small vibrations. And all the calculated elastic constants meet the mechanical stability criteria for an orthorhombic system (Wu *et al.*, 2007 ▸), indicating that *Pbam*48 and *Pbam*40 possess positive mechanical stabilities to resist minor deformations as carbon and silicon. Based on the calculated elastic constants, we can effectively evaluate the mechanical properties (Wu *et al.*, 2007 ▸) of *Pbam*48 and *Pbam*40 as carbon and silicon. The calculated bulk modulus (*B*), shear modulus (*G*), Young’s modulus (*Y*), Poisson’s ratio (ν) and Vicker’s hardness (*Hv*) (Niu *et al.*, 2011 ▸) are summarized in Tables S5 and S6. The results suggest that, as carbon allotropes, both *Pbam*48 (85.35 GPa) and *Pbam*40 (85.56 GPa) are superhard materials with hardness comparable to c-diamond (92.36 GPa). The hardness values of *Pbam*48 and *Pbam*40 as silicon are only 11.38 and 11.06 GPa, respectively, suggesting that they can be as easily machined into various circuit patterns as diamond silicon with a hardness of 11.5 GPa.

**Figure 5:**
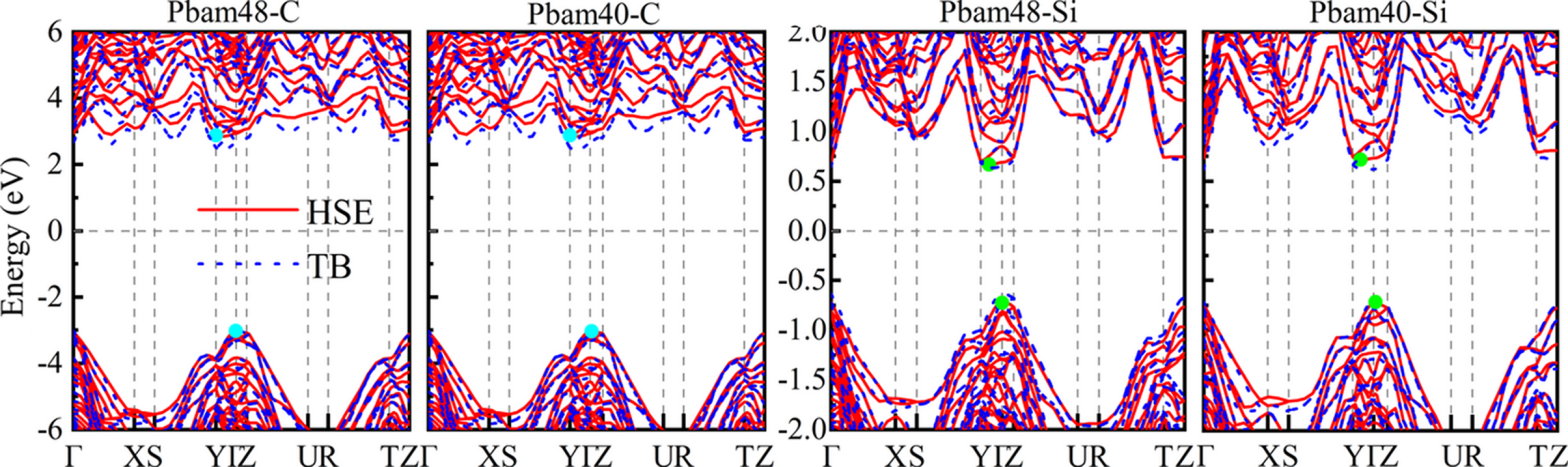
Electronic band structures of *Pbam*48 and *Pbam*40 as carbon and silicon calculated from the HSE06 functionals (red solid lines) and TB model (blue dashed lines).

According to our tight binding (TB) based high-throughput results shown in Fig. S5, both *Pbam*48 and *Pbam*40 identified carbon to be an insulator and silicon to be a semiconductor. To further validate the accuracy of the TB-based results, we calculated their HSE06-based band structures for comparison. As shown in Fig. 5 ▸, the TB-based results align well with the HSE06-based results in both carbon and silicon systems, with both the band gap types and the sizes matching closely. This further demonstrates the reliability of the TB parameters we used for carbon and silicon. The HSE06-based results show that *Pbam*48 and *Pbam*40, as carbon, are insulators with quasi-direct band gaps of 5.622 and 5.890 eV, respectively. These band gaps are larger than that of c-diamond (5.32 eV), but still smaller than that of the widest band gap carbon of I-43d (7.25 eV) (He *et al.*, 2018 ▸). As silicon, both *Pbam*48 and *Pbam*40 can be identified as quasi-direct band gap semiconductors. Their smallest direct band gaps are 1.407 and 1.468 eV, which are just slightly larger than the smallest indirect band gaps of 1.386 and 1.451 eV, respectively. These values of band gaps indicate that *Pbam*48-Si and *Pbam*40-Si are potential materials for application in solar cells as absorbers.

## 4. Conclusions

In summary, our study systematically investigates the structural and electronic properties of 4-coordinated post-graphite candidates using a graph-theoretical approach. We demonstrate that these structures can be decomposed into unique (2+1)-regular bipartite-like graphs, offering a novel method for generating 3D 4-coordinated networks. The random method based on group and graph theory (RG2) proves highly effective, yielding numerous (2+1)-regular bipartite-like graphs for constructing potential carbon and silicon phases. Among the identified phases, *Pbam*48 and *Pbam*40 stand out. As carbon allotropes, they exhibit superhardness exceeding 85 GPa and wide quasi-direct band gaps (5.622 and 5.890 eV), making them ideal for applications requiring high hardness and insulation. As silicon phases, their quasi-direct band gaps (1.386 and 1.451 eV) suggest suitability as efficient solar cell absorber materials. These phases also demonstrate superior or comparable energetic stability to the previously proposed *Pbam*24 structure as carbon and the experimentally synthesized Si-24 as silicon, reinforcing their viability as stable carbon/silicon phases. These findings not only enhance our understanding of post-graphite phases, but also introduce promising materials for semiconductor and mechanical applications, emphasizing the power of graph-theoretical methods in materials design.

## Supplementary Material

Supporting figures and tables. DOI: 10.1107/S2052252525005810/zx5033sup1.pdf
