# Supplementary material for: Low-energy tetrahedral networks for carbon and silicon from (2+1)-regular bipartite-like graphs
Source: IUCrJ. 2025 Jul 29;12(Pt 5):523–30. doi: 10.1107/S2052252525005810 (PMC12403172; doi:10.1107/S2052252525005810)
Supplement: Supplementary file 1 [file m-12-00523-sup1.pdf]

IUCrJ

Volume 12 (2025)

Supporting information for article:

Low-energy tetrahedral networks for carbon and silicon from (2+1)-regular bipartite-like graphs

Yalan Wei, Shifang Li, Xizhi Shi and Chaoyu He

Contents

S1. Supplementary figures:

Fig.S1 An illustration of converting 2D (n+m)-regular bipartite-like graph with AB stacking into 3D (n+(m+1))-regular bipartite-like graph.

Fig.S2 Decompositions of M-carbon, W-carbon and S-carbon along different directions into graphite or non-graphite layers. 4-regular graphs of M-carbon and W-carbon contains (0+3) and (2+1)-regular bipartite-like graphs, while S-carbon contains hybrid (0+3) and (2+1) non-bipartite graphs. All these three structures can be decomposed into non-graphite layers, which can be identified as (2+1)-regular bipartite-like graphs.

Fig.S3 Examples for (m+n)-regular bipartite graphs generated by RG2. (a), (b) and (c) are (0+3)-, (1+2)- and (2+1)-regular bipartite-like graphs, where we selected one example to demonstrate the effects of AA and AB stacking for each type.

Fig.S4 Band structures of the fitting (CFS, c-diamond, BC8, T12) and validation (R8, Pbam24, St12.) sets based on TB (blue dash lines) parameters fitted with HSE06 (red solid lines).

Fig.S5 Scatter plots of the PBE-based relative average energies (eV/atom) versus the TB-based band gaps (Eg/eV) of the discovered carbon (a) and silicon (b) allotropes. Q, D and I indicate the quasi-direct, direct and indirect band gap semiconductors, respectively.

Fig.S6 The simulated X-ray diffraction (XRD) patterns for the Pbam48 and Pbam40 as carbon in comparison with the widely investigated M-carbon, Z-carbon, W-carbon, H-carbon, S-carbon and Pbam24, as well as the experimental XRD data of the cold-compressed graphite at 23.9 and 13.7 GPa.

Fig.S7 The simulated vibrational spectrums for the Pbam48 and Pbam40 structures as carbon and silicon.

S2. Supplementary tables:

Table S1: Tight-Binding Parameters for Carbon with sp^3d^5 Basia[a]

Table S2: Crystalline information for Pbam48 and Pbam40 as carbon

Table S3: Crystalline information for Pbam48 and Pbam40 as silicon

Table S4: The calculated elastic constants (C_{ij}: GPa), bulk modulus (B: GPa), shear modulus (G: GPa), Yang's modulus (Y: GPa) and Poisson ration(v: 1) for the Pbam48 and Pbam40 as carbon and silicon.

Table S5: The PBE-based total energies (E_{tot}: meV/atom) relative to the corresponding c-diamond form, equilibrium volume per atom (V_0: Å^3/atom) and the energy band gaps (eV) calculated based on TB and HSE06, as well as the and Vicker hardness (H_v: GPa) for various typical configurations of carbon and silicon.

Table S6 The optimized lattice constants (Å), the corresponding Brillouin Zone sample meshes, enegy cutoffs (eV) and all convergence criteria (used for the relaxtion structure and DFPT for the calucation of elastic constants and phonon properties).

S1. Supplementary figures:

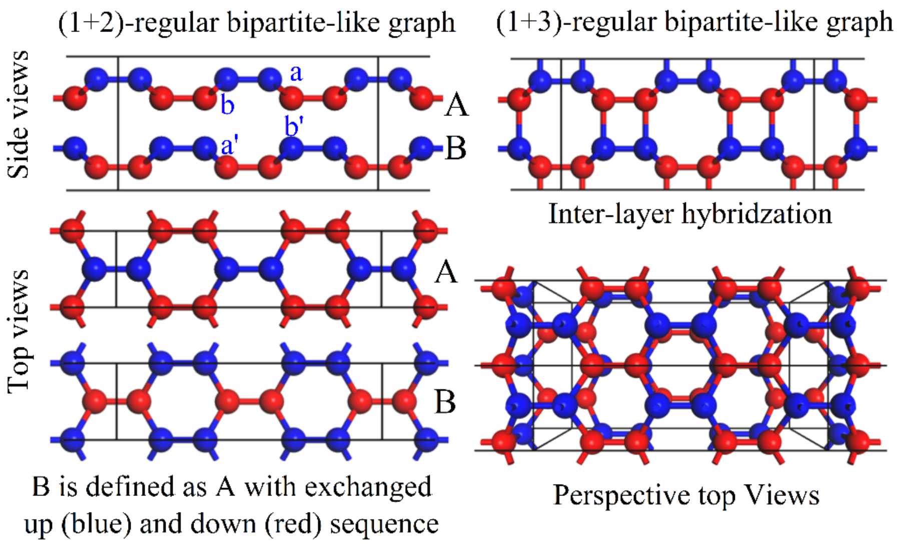

Fig. S1 An illustration of converting 2D (n+m)-regular bipartite-like graph with AB stacking into 3D (n+(m+1))-regular bipartite-like graph.

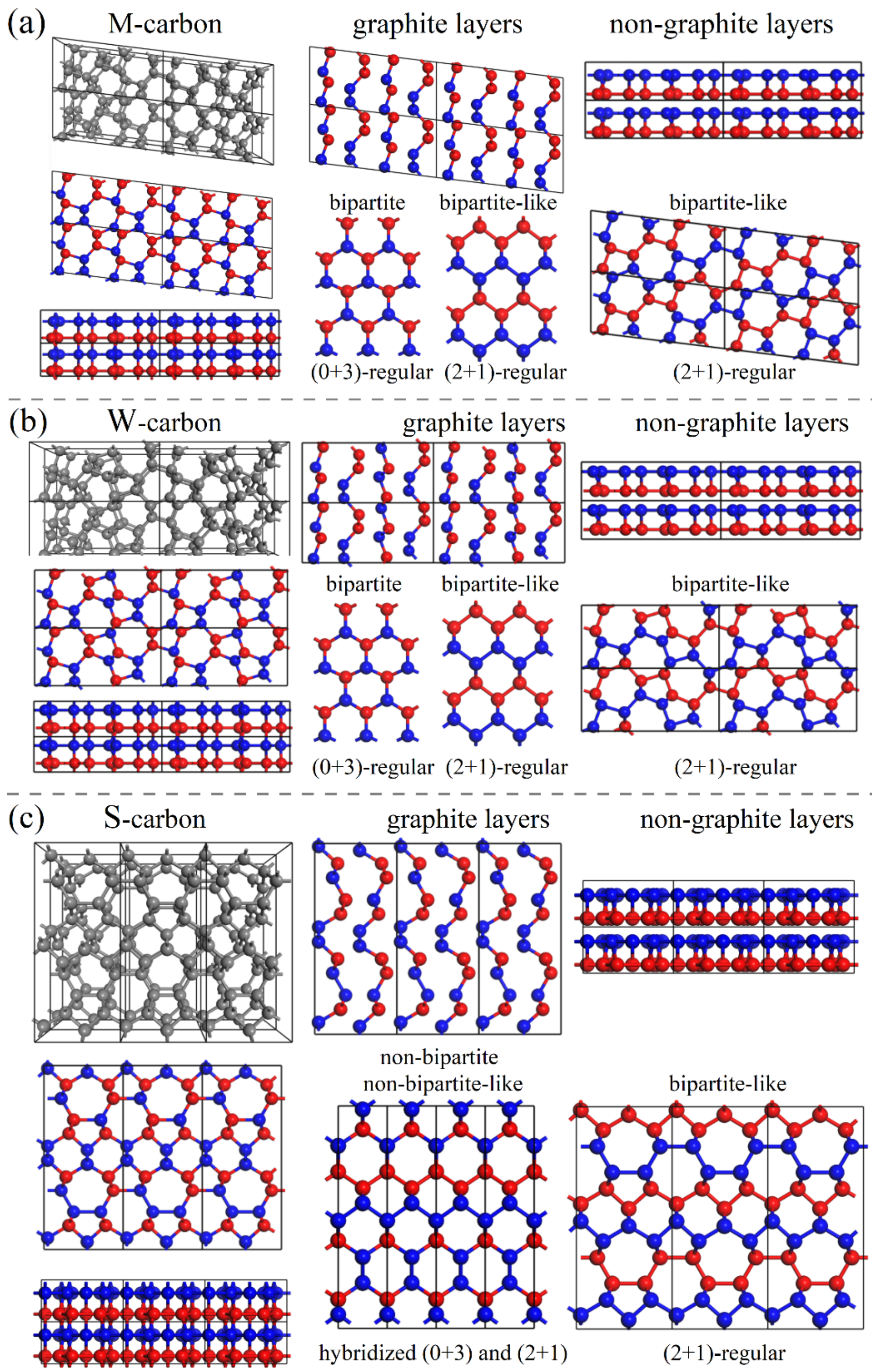

Fig.S2 Decompositions of M-carbon, W-carbon and S-carbon along different directions into graphite or non-graphite layers. 4-regular graphs of M-carbon and W-carbon contains (0+3) and (2+1)-regular bipartite-like graphs, while S-carbon contains hybrid (0+3) and (2+1) non-bipartite graphs. All these three structures can be decomposed into non-graphite layers, which can be identified as (2+1)-regular bipartite-like graphs.

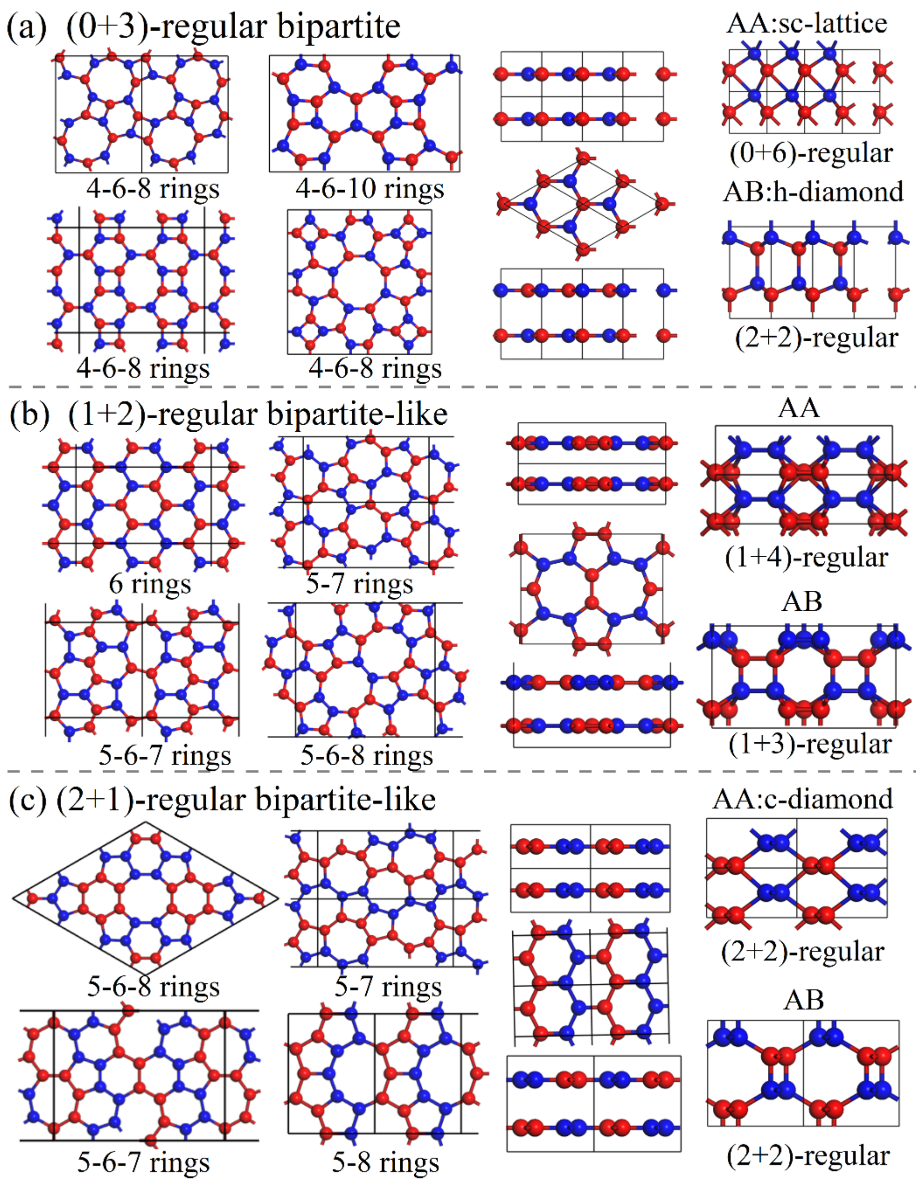

Fig.S3 Examples for (m+n)-regular bipartite graphs generated by RG2. (a), (b) and (c) are (0+3)-, (1+2)- and (2+1)-regular bipartite-like graphs, where we selected one example to demonstrate the effects of AA and AB stacking for each type.

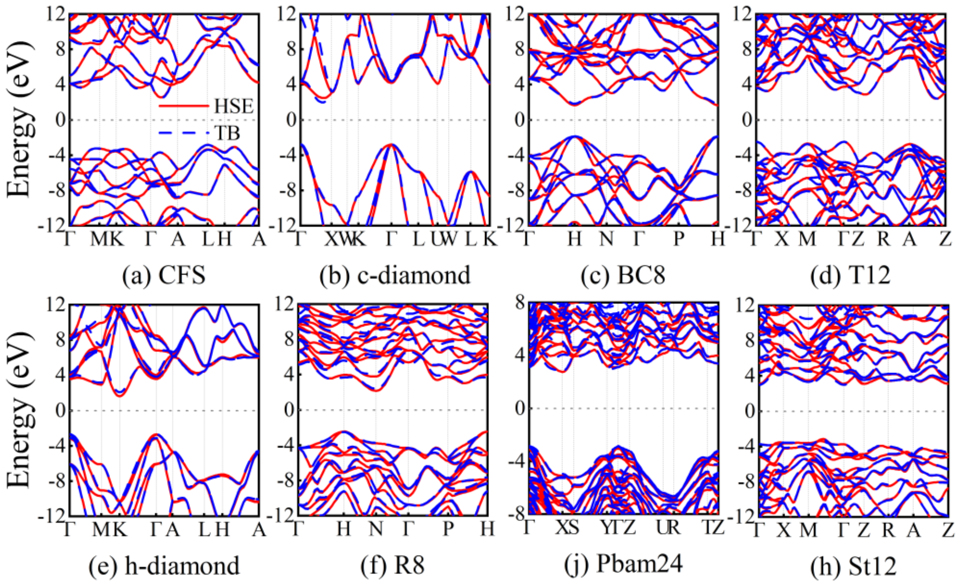

Fig. S4 Band structures of the fitting (CFS, c-diamond, BC8, T12) and validation (R8, Pbam24, St12.) sets based on TB (blue dash lines) parameters fitted with HSE06 (red solid lines).

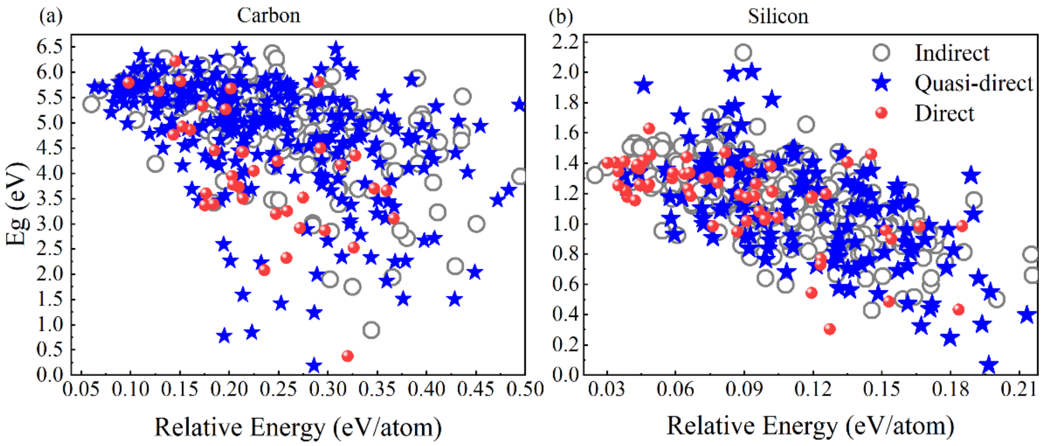

Fig. S5 Scatter plots of the PBE-based relative average energies (eV/atom) versus the TB-based band gaps (Eg/eV) of the discovered carbon (a) and silicon (b) allotropes. Q, D and I indicate the quasi-direct, direct and indirect band gap semiconductors, respectively.

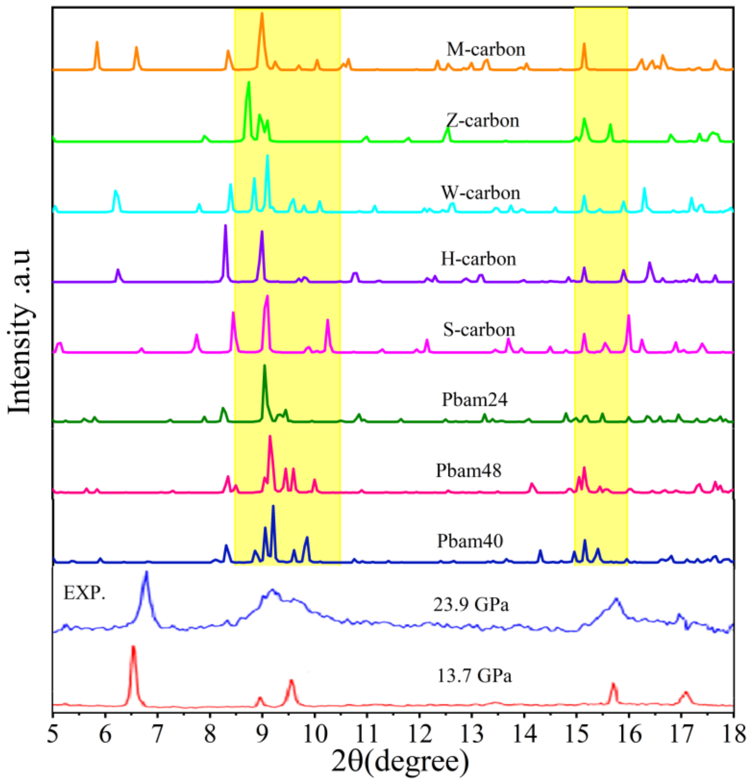

Fig. S6 The simulated X-ray diffraction (XRD) patterns for the Pbam48 and Pbam40 as carbon in comparison with the widely investigated M-carbon, Z-carbon, W-carbon, H-carbon, S-carbon and Pbam24, as well as the experimental XRD data of the cold-compressed graphite at 23.9 and 13.7 GPa.

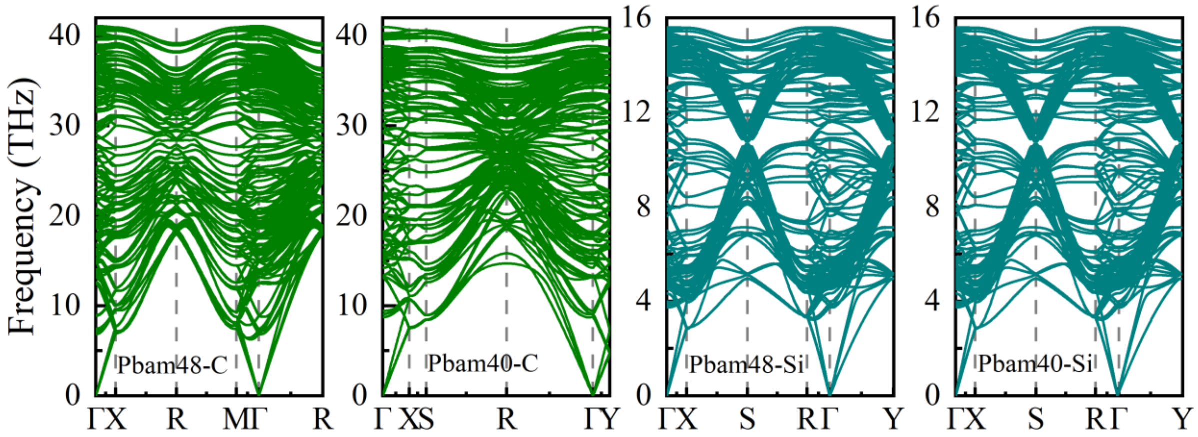

Fig. S7 The simulated vibrational spectrums for the Pbam48 and Pbam40 structures as carbon and silicon.

S2. Supplementary tables:

Table S1: Tight-Binding Parameters for Carbon with sp^3d^5 Basia[a]

		$ss\sigma$	$sp\sigma$	$pp\sigma$	ppπ	$sd\sigma$	$pd\sigma$	$pd\pi$	$dd\sigma$	$dd\pi$	$dd\delta$
t	V	-6.0487	6.261	5.662	-3.000	3.923	3.964	2.440	13.779	-3.727	-0.228
	q1	2.145	2.283	2.127	3.334	12.950	6.313	2.5134	3.696	6.121	8.106
s	S	0.369	-0.362	-0.371	0.1554	0.327	0.191	-0.145	0.291	-0.132	0.060
	q2	2.156	1.959	2.015	2.409	1.289	1.221	1.616	2.461	1.137	0.726
E		E_s=1.426		E_P=13.662		E_d=31.762					

[a]Units of on-site energies and hopping integrals are in electronvolts

Table S2: Crystalline information for Pbam48 and Pbam40 as carbon.

Name:Pbam48-C, Space group:55(Pbam), Lattice: a=7.638 Å, b=14.411 Å, c=2.524 Å; α=β=γ=90°							
C1	0.95390	0.26278	0.00000	C7	0.93253	0.71906	0.50000
C2	0.69634	0.49437	0.00000	C8	0.36254	0.88133	0.50000
C3	0.64099	0.92879	0.00000	C9	0.46873	0.62663	0.50000
C4	0.24883	0.40055	0.00000	C10	0.18526	0.19301	0.50000
C5	0.20532	0.67563	0.00000	C11	0.47151	0.05225	0.50000
C6	0.57383	0.65344	0.00000	C12	0.58600	0.47291	0.50000
Name: Pbam40-C, Space group:55(Pbam), Lattice: a=7.654 Å, b=12.026 Å, c=2.525 Å; α=β=γ=90°							
C1	0.09512	0.68114	0.00000	C6	0.4205	0.95889	0.50000
C2	0.75193	0.40077	0.00000	C7	0.97208	0.06664	0.50000
C3	0.71129	0.71467	0.00000	C8	0.14792	0.13134	0.50000
C4	0.69204	0.02038	0.00000	C9	0.01437	0.33686	0.50000
C5	0.86074	0.09366	0.00000	C10	0.8258	0.73667	0.50000

Table S3: Crystalline information for Pbam48 and Pbam40 as silicon.

Name:Pbam48-Si, Space group:55(Pbam), Lattice: a=11.670 Å, b=21.967 Å, c=3.864 Å; α=β=γ=90°							
C1	0.95503	0.262178	0.00000	C7	0.93234	0.71892	0.50000
C2	0.69727	0.49547	0.00000	C8	0.36479	0.88237	0.50000
C3	0.64130	0.92901	0.00000	C9	0.47016	0.62747	0.50000
C4	0.24726	0.40023	0.00000	C10	0.18638	0.19428	0.50000
C5	0.20507	0.67425	0.00000	C11	0.47172	0.05215	0.50000
C6	0.57617	0.65387	0.00000	C12	0.58760	0.47389	0.50000
Name:Pbam40-Si, Space group:55(Pbam), Lattice: a=11.707 Å, b=18.313 Å, c=3.864 Å; α=β=γ=90°							
C1	0.42135	0.95881	0.50000	C7	0.09542	0.68201	0.00000
C2	0.9714	0.06437	0.50000	C8	0.75054	0.40017	0.00000
C3	0.14584	0.13203	0.50000	C9	0.71130	0.71361	0.00000
C4	0.01375	0.33705	0.50000	C10	0.69114	0.02176	0.00000
C5	0.82452	0.73762	0.50000	C11	0.86057	0.0923	0.00000

Table S4. The calculated elastic constants (C_{ij}: GPa), bulk modulus (B: GPa), shear modulus (G: GPa), Yang's modulus (Y:GPa) and Poisson ration(v: 1) for the Pbam48 and Pbam40 as carbon and silicon.

System	C_{11}	C_{22}	C_{33}	C_{44}	C_{55}	C_{66}	C_{12}	C_{13}	C_{23}	B	G	Y	P
Pbam48-C	1081	1160	1183	466	548	503	140	126	56	452	510	1112	0.090
Pbam40-C	1029	1087	1122	441	526	476	125	104	42	420	486	1053	0.082
Pbam48-Si	154	178	175	48	67	61	51	51	32	86	60	146	0.217
Pbam40-Si	154	176	174	46	67	60	51	52	32	86	59	144	0.219

Table S5. The PBE-based total energies (E_{tot}: meV/atom) relative to the corresponding c-diamond form, equilibrium volume per atom (V_0:Å^3/atom) and the energy band gaps (eV) calculated based on TB and HSE06, as well as the and Vicker hardness (H_v:GPa) for various typical configurations of carbon and silicon.

System	E_{tot}	V_0	E_g^{TB}	E_g^{HSE}	H_v	Gap type
c-diamond	0	5.65	5.13	5.32	92.36	I
h-diamond	25	5.66	4.76	*3.29*	95.43	I
V-carbon	105	5.79	4.56	4.44	-	I
I-43d	112	5.74	7.23	7.25	85.33	D
Pbam24-C	68	5.81	5.705	5.840	83.86	I
Pbam48-C	60	5.792	5.368	5.622	85.35	Q
Pbam40-C	69	5.812	5.636	5.890	85.56	Q
c-diamond	0	20.44	1.03	1.19	12.51	I
h-diamond	11	20.41	1.08	1.110	11.99	I
Si-24	92	21.99	1.26	1.07	-	Q
I-43d	39	20.44	2.05	2.19	10.26	Q
Pbam24-Si	29	20.97	1.401	1.38	9.81	D
Pbam48-Si	24.218	20.634	1.277	1.386	11.38	Q
Pbam40-Si	29.166	20.708	1.334	1.451	11.06	Q

Table S6 The optimized lattice constants (Å) , the corresponding Brillouin Zone sample meshes , enegy cutoffs (eV) and all convergence criteria(used for the relaxtion structure and DFPT for the calucation of elastic constants and phonon properties).

	Lattice (Å)	Meshe	Enengy cutoffs	Force citeria	DFPT (force citeria)
Pbam48-C	a=7.638, b=14.411, c=2.524	4×3×12	500	0.01	0.0001
Pbam40-C	a=7.654, b=12.026, c=2.525	4×3×12	500	0.01	0.0001
Pbam48-Si	a=11.670, b=21.967, c=3.864	3×2×9	400	0.01	0.0001
Pbam40-Si	a=11.707, b=18.313, c=3.864	3×2×9	400	0.01	0.0001

Amsler, M., Flores-Livas, J. A., Lehtovaara, L., Balima, F., Ghasemi, S. A., Willand, A., Caliste, D., Goedecker, S. & Marques, M. A. (2012). *Physical Review Letters* **108**, 065501.

Biswas, R., Martin, R. M., Needs, R. J. & Nielsen, O. H. (1987). *Physical Review B* **35**, 9559-9568.

Bj□orn Winkle, Chris J. Pickard, Victor Milman & c, G. T. (2001). *Chemical Physics Letters* **337**, 36.

Blochl, P. E. (1994). *Phys Rev B Condens Matter* **50**, 17953-17979.

El Goresy, A., Dubrovinsky, L. S., Gillet, P., Mostefaoui, S., Graup, G., Drakopoulos, M., Simionovici, A. S., Swamy, V. & Masaitis, V. L. (2003). *Comptes Rendus Géoscience* **335**, 889.

Glass, C. W., Oganov, A. R. & Hansen, N. (2006). *Computer Physics Communications* **175**, 713-720.

GoRESY, A. E. & DONNAY, G. (1968). *Scicence* **161**, 363-364.

Hawfhorne, F. C. (1990). *Nature* **345**, 297.

He, C., Shi, X., Clark, S. J., Li, J., Pickard, C. J., Ouyang, T., Zhang, C., Tang, C. & Zhong, J. (2018). *Physical Review Letters* **121**, 175701.

He, C., Sun, L., Zhang, C., Peng, X., Zhang, K. & Zhong, J. (2012). *Solid State Communications* **152**, 1560-1563.

He, C., Zhang, C. X., Xiao, H., Meng, L. & Zhong, J. X. (2017). *Carbon* **112**, 91-96.

Heyd, J. & Scuseria, G. E. (2003). *Journal of Chemical Physics* **118**, 8207-8215.

Iijima, S. (1991). *Nature* **354**, 56-58.

Kim, D. Y., Stefanoski, S., Kurakevych, O. O. & Strobel, T. A. (2015). *Nature Materials* **14**, 169-173.

Kresse, G. & Furthmu¨ller, J. (1966). *Physical Review B* **54**, 11169.

Kresse, G. & Joubert, D. (1999). *Physical Review B* **59**, 1758.

Kroto, H. W., Heath, J. R., O'Brien, S. C., Curl, R. F. & Smalley, R. E. (1985). *Nature* **318**, 162.

Lee, I.-H., Lee, J., Oh, Y. J., Kim, S. & Chang, K. J. (2014). *Physical Review B* **90**, 115209.

Li, C., Liang, H., Zhang, X., Lin, Z. & Wei, S. (2023). *npj Computational Materials* **9**, 176.

Li, D., Bao, K., Tian, F., Zeng, Z., He, Z., Liu, B. & Cui, T. (2012). *Phys Chem Chem Phys* **14**, 4347-4350.

Li, Q., Ma, Y., Oganov, A. R., Wang, H., Wang, H., Xu, Y., Cui, T., Mao, H. K. & Zou, G. (2009). *Physical review letters* **102**, 175506.

Liao, Y., Shi, X., Luo, C. & He, C. (2023). *Materials Advances* **4**, 709-714.

Liao, Y., Shi, X., Ouyang, T., Li, J., Zhang, C., Tang, C., He, C. & Zhong, J. (2021). *J. Phys. Chem. Lett.* **12**, 8889-8896.

Lonie, D. C. & Zurek, E. (2011). *Computer Physics Communications* **182**, 372-387.

Mao, W. L., Mao, H. K., Eng, P. J., Trainor, T. P. & Hemley, R. J. (2003). *Science* **302**, 425-427.

Mujica, A., Pickard, C. J. & Needs, R. J. (2015). *Physical Review B* **91**, 214104.

Niu, H., Chen, X. Q., Wang, S., Li, D., Mao, W. L. & Li, Y. (2012). *Physical Review Letters* **108**, 135501.

Niu, H., Wei, P., Sun, Y., Chen, X.-Q., Franchini, C., Li, D. & Li, Y. (2011). *Applied Physics Letters* **99**, 031901.

Novoselov, K. S., Geim, A. K., Morozov, S. V., Jiang, D., Zhang, Y., Dubonos, S. V., Grigorieva, I. V. & Firsov, A. A. (2004). *Science* **306**, 666-669.

Oganov, A. R., Pickard, C. J., Zhu, Q. & Needs, R. J. (2019). *Nature Reviews Materials* **4**, 331-348.

Pickard, C. J. & Needs, R. J. (2010). *Physical Review B* **81**, 014106.

Pickard, C. J. & Needs, R. J. (2011). *J Phys Condens Matter* **23**, 053201.

Piltz, R. O., Maclean, J. R., Clark, S. J., Ackland, G. J., Hatton, P. D. & Crain, J. (1995). *Phys Rev B* **52**, 4072-4085.

Rapp, L., Haberl, B., Pickard, C. J., Bradby, J. E., Gamaly, E. G., Williams, J. S. & Rode, A. V. (2015). *Nature Communications* **6**, 7555.

Rulong Zhou & Zeng, X. C. (2012). *J Am Chem Soc* **134**, 7530.

SACADA.

Sheng, X. L., Yan, Q. B., Ye, F., Zheng, Q. R. & Su, G. (2011). *Physical Review Letters* **106**, 155703.

Shi, X., He, C., Pickard, C. J., Tang, C. & Zhong, J. (2018). *Physical Review B* **97**, 014104

Strong, R. T., Pickard, C. J., Milman, V., Thimm, G. & Winkler, B. (2004). *Physical Review B* **70**, 045101.

Su, L., Li, S., Li, J., He, C., Ouyang, T., Chunxiao, Z., Tang, C. & Zhong, J. (2022). *ACS Materials Letters* **4**, 1726-1733.

Takagi, M., Taketsugu, T., Kino, H., Tateyama, Y., Terakura, K. & Maeda, S. (2017). *Physical Review B* **95**, 184110.

Togo, A. & Tanaka, I. (2015). *Scripta Materialia* **108**, 1-5.

Umemoto, K., Wentzcovitch, R. M., Saito, S. & Miyake, T. (2010). *Physical review letters* **104**, 125504.

Wang, J., Gao, H., Han, Y., Ding, C., Pan, S., Wang, H.-T., Xing, D. & Sun, J. (2023). *National Science Review* **10**, nwad128.

Wang, J. T., Chen, C. & Kawazoe, Y. (2011). *Physical Review Letters* **106**, 075501.

Wang, Y., Lv, J., Zhu, L. & Ma, Y. (2010). *Physical Review B* **82**, 094116.

Wang, Y., Lv, J., Zhu, L. & Ma, Y. (2012). *Computer Physics Communications* **183**, 2063-2070.

Wei, Y., Shi, X., Li, J. & He, C. (2022). *Scripta materialia* **219**, 114843.

Whittaker, A. g. & Kintner, P. L. (1969). *Science* **165**, 589-591.

Wolten, A. G. W. a. G. M. (1972). *Science* **178**, 54-56.

Woodley, S. M. & catlow, R. (2008). *Nature materials* **7**, 937-946.

Wu, X., Vanderbilt, D. & Hamann, D. R. (2005). *Physical Review B* **72**, 035105.

Wu, Z., Zhao, E., Xiang, H., Hao, X., Liu, X. & Meng, J. (2007). *Physical Review B* **76**, 054115.

Xie, T. & Grossman, J. C. (2018). *Physical Review Letters* **120**, 145301.

Yamada, K. (2003). *Carbon* **41**, 1309-1313.

Yang, X., Yao, M., Wu, X., Liu, S., Chen, S., Yang, K., Liu, R., Cui, T., Sundqvist, B. & Liu, B. (2017). *Physical Review Letters* **118**, 245701.

Yin, H., Shi, X., He, C., Li, J., Pickard, C. J., Tang, C., Ouyang, T., Zhang, C. & Zhong, J. (2019). *Physical Review B* **99**, 041405(R).

Zahariev, F., Dudiy, S. V., Hooper, J., Zhang, F. & Woo, T. K. (2006). *Physical review letters* **97**, 155503.

Zhang, J., Wang, R., Zhu, X., Pan, A., Han, C., Li, X., Wang, W., Su, H. & Niu, C. (2017). *Nature Communications* **8**, 683.

Zhang, M., He, C. & Zhong, J. (2024). *Journal of Physics D: Applied Physics* **57**, 385109.

Zhao, Z., Tian, F., Dong, X., Li, Q., Wang, Q., Wang, H., Zhong, X., Ma, Y. & Tian, Y. (2012). *Journal of the American Chemical Society* **134**, 12362−12365.

Zhao, Z., Xu, B., Zhou, X. F., Wang, L. M., Wen, B., He, J., Liu, Z., Wang, H. T. & Tian, Y. (2011). *Physical Review Letters* **107**, 215502.

Zhong, Y., Yu, H., Su, M., Gong, X. & Xiang, H. (2023). *npj Computational Materials* **9**, 182.
